# Multiple uterine perforations during manual vacuum aspiration: the need to increase the clinical awareness of attending healthcare professionals

**DOI:** 10.4314/ahs.v22i1.23

**Published:** 2022-03

**Authors:** Nnabuike C Ngene

**Affiliations:** 1 Department of Obstetrics and Gynaecology, School of Clinical Medicine, Faculty of Health Sciences, University of the Witwatersrand, Johannesburg, South Africa; 2 Department of Obstetrics and Gynaecology, Klerksdorp Hospital, Klerksdorp, South Africa

**Keywords:** Image of uterine perforation, manual vacuum aspiration, uterine sounding

## Abstract

**Background:**

The risk of uterine perforation during manual vacuum aspiration (MVA) is reduced by using Karman cannula (which has a rounded tip) during the procedure.

**Methods:**

A 35-year-old multigravida at 13 gestational weeks presented with vaginal bleeding of a day duration and ultrasound evidence of retained products of conception suggestive of incomplete miscarriage. The patient was rhesus D positive and stable. She had MVA which was performed using Karman cannula, and developed severe vaginal bleeding. The differential diagnoses were incomplete uterine evacuation and uterine perforation.

**Results:**

During a laparotomy in Lloyd-Davies position, haemoperitoneum and six uterine perforations on the anterior and fundal parts, each approximately 5 mm in length (Figure 1), were found. The perforations were repaired and a check uterine curettage under oxytocic cover showed an empty uterus. The abdominal cavity was washed and closed. She was transfused three units of red blood cell concentrate and had a normal six weeks follow-up.

**Conclusion:**

When an instrument inserted into the uterus is pushed beyond the estimated depth of the uterus, a perforation must be suspected and the condition may be managed conservatively. A surgical procedure complicated by surgeon's loss of perception (in this case tactile) of tissues' anatomy is hazardous.

## Background

As many as 10 – 15% of clinically recognized pregnancies end as miscarriages^1^,^2^ and many of the patients will require surgical uterine evacuation using manual vacuum aspiration (MVA). Sounding the uterus during such a procedure may be useful but has a risk of perforation. The risk of uterine perforation during MVA is reduced by using Karman cannula (which has a rounded tip) but the complication may still occur and result in a laparotomy in severe cases. During the first trimester surgical abortion, the risk of uterine perforation is 0.1 to 4 per 1000 procedures 3, 4. The instruments that may perforate the uterus during the procedure, in decreasing order of risk occurrence, are suction cannula, uterine sound and cervical dilators^5^. Regardless, the risk of perforation increases with the inexperience of the operator, lack of cervical priming/dilatation, the advancement of weeks of gestation, retroverted uterus and previous delivery. Interestingly, a high-quality image of multiple “simple/silent” uterine perforations resulting from the use of Karman cannula that necessitated laparotomy is scarce to find, and the index report provides the information and learning lessons.

## Methods

A 35-year-old multigravida at 13 gestational weeks presented with vaginal bleeding of a day duration and ultrasound evidence of retained products of conception suggestive of incomplete miscarriage. The patient was rhesus D positive and stable. She had MVA which was performed using Karman cannula without any uterotonic agent administered before or during the procedure. Before the insertion of the cannula into the uterine cavity, bimanual pelvic examination confirmed an open cevical os and an anteverted 12 weeks' size uterus. A Cusco's speculum was then inserted into the vagina to expose the cervix. The depth of the uterus was estimated to be 12 cm by using a vulsellum forceps to maintain gentle traction on the anterior lip of the cervix while a calibrated Sims uterine sound was introduced into the uterine cavity up until the maximum depth of the uterus to read-off the graduation at the level of the external cervical os. The uterine sounding was easy and performed only once. During the aspiration period, she developed severe vaginal bleeding. The differential diagnoses were incomplete uterine evacuation and uterine perforation.

## Results

**Figure 1:**
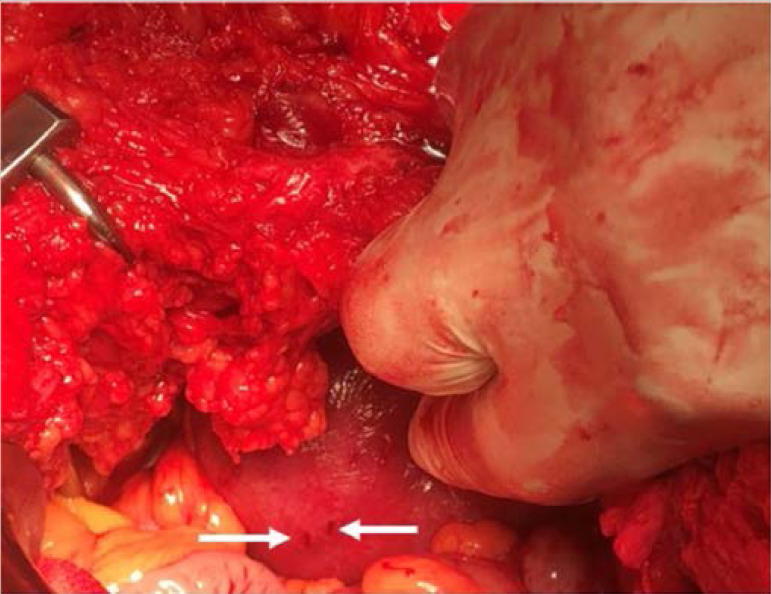
Uterine perforations (arrows) with Karman cannula during manual vacuum aspiration.

During a laparotomy in Lloyd-Davies position, haemoperitoneum and six uterine perforations on the anterior and fundal parts, each approximately 5 mm in length (Figure 1), were found. The perforations were repaired and a check uterine curettage under oxytocic cover showed an empty uterus. The abdominal cavity was washed and closed. She was transfused three units of red blood cell concentrate and had a normal six weeks follow-up.

## Conclusion

Administration of a uterotonic agent such as misoprostol before a MVA facilitates priming of the cervix, uterine contraction and tone, and improves recognition of uterine fundus. The use of uterine sound functions to determine the cervical/uterine anatomy and in some cases dilate the cervix. While uterine sounding may be helpful it also carries a risk of perforation, particularly when the uterus is not contracted and if the instrument used for sounding has a pointed rather than a broad/blunt-ending tip. If there is difficulty in determining the uterine anatomy, assistance from an experienced clinician should be sort and the use of cervical dilators to dilate closed cervical os) and pelvic ultrasonography may also be helpful. Due to the risk of perforation, direct sounding with a uterine sound may be omitted by some healthcare professionals (particularly when the uterus is not contracted) but performed indirectly with a blunt-ending instrument during the procedure. If sounding has been omitted and or difficult, it is a good clinical practice for the uterine position and cavity length to be assessed during the MVA, preferably using ultrasonography. Importantly, evacuation of the uterus under uterotonic cover improves uterine contraction and prevents perforation. Of note, the perforations in the index case resulted from the use of Karman cannula and not from uterine sounding which was performed only once. However, when an instrument inserted into the uterus is pushed beyond the estimated depth of the uterus, a perforation must be suspected and the condition may be managed conservatively given the minimal tissue damage of a single simple perforation (Figure 1). Failure to recognize the injury may result in multiple perforations and excessive bleeding requiring a laparotomy. While the anterior and fundal parts of the uterus are the commonest sites of uterine perforation, the injury may occur at different locations on the uterus, cervix and vaginal fornix. Occasionally, a perforation by Karman cannula may result in abdominopelvic viscera such as the omentum and the small intestine being sucked into the aperture on the distal end of the cannula and a loop of bowel can be pulled into the vagina and possibly beyond the introitus with consequential tissue damage. To prevent evisceration of the bowel and associated tissue damage, the pressure in the MVA set must be released before the withdrawal of the cannula from the genitalia if perforation is suspected. Notably, a surgical procedure complicated by surgeon's loss of perception (in this case tactile) of tissues' anatomy is hazardous.
